# Evaluation of a Depression Intervention in People With HIV and/or TB in Eswatini Primary Care Facilities: Implications for Southern Africa

**DOI:** 10.9745/GHSP-D-22-00016

**Published:** 2023-04-28

**Authors:** Nina Putnis, Nick Riches, Archibald Nyamayaro, Darah Boucher, Rebecca King, Ian F. Walker, Alett Burger, Paul Southworth, Violet Mwanjali, John Walley

**Affiliations:** a Nuffield Institute of International Health and Development, University of Leeds, Leeds, United Kingdom.; b Good Shepherd Hospital, Siteki, Eswatini.; c Lubombo Health Research Unit, Siteki, Eswatini.; d National Psychiatric Referral Hospital, Manzini, Eswatini.

## Abstract

**Introduction:**

Depression associated with chronic illnesses is common in Southern Africa, yet there are major treatment gaps. This study assesses the feasibility and acceptability of the Healthy Activity Program intervention for depression among people with HIV and/or TB. The intervention involves training nonspecialist nurses in depression, including identification, counseling based on behavioral activation theory, and structured referral.

**Methods:**

This is a mixed methods evaluation of a pilot counseling service integrated within routine HIV and TB care from 2018 to 2019. Participants included people living with HIV and/or patients with TB in rural Eswatini.

**Results:**

A total of 324 people living with HIV and/or TB were screened for depression, with 19% (62/324) screening positive. The median number of sessions attended was 3 (interquartile range: 1–5), with 16/60 (26%) attending the minimum 5 sessions. Qualitative results indicated acceptability, but there were concerns about feasibility.

**Conclusions:**

The Healthy Activity Program is a promising option to manage the treatment gap for depression in people with HIV and/or TB. However, task-shifting to nonspecialist health care professionals without increasing staff capacity is a barrier to implementation. Realistic and pragmatic assessments of capacity and workforce are essential.

## Abstract (precis)

The authors assessed the feasibility and acceptability of a program integrating basic mental health care into nurse-led HIV and TB care in rural settings in Eswatini and identified key barriers to implementation and scale-up.

## Teaser

**Key Findings:**

- Integrating care for chronic diseases, including mental health conditions, is a critical yet challenging element of universal health coverage goals, especially in low- and middle-income countries. The simple intervention for depression and anxiety, based on behavioral methodologies, supports people living with HIV and/or TB with comorbid depression or anxiety.
- This pilot service evaluation presents real-life findings, with a focus on acceptability and feasibility, demonstrating issues around feasibility and the capacity of nonspecialist nursing staff to undertake additional mental health counseling without explicit support and resources, including time. It demonstrates acceptability among staff and patients.

**Key Implications:**

- Policymakers, health systems leaders, and health service staff should recognize the importance of integrated care, especially neglected mental health care, and advocate for dedicated resources, time, and capacity of health workers to take on this additional role.
- Meaningful consultation with the health workforce is a critical element of service development.

## INTRODUCTION

Depression is the most common mental health condition worldwide, including in sub-Saharan Africa (SSA).^1^ Estimated prevalence of mental disorders in low- and middle-income countries is high, yet data are scant, and mental health systems are limited and underresourced.^2^^–^^4^ The World Health Organization’s Mental Health Gap Action Program outlines that the majority of people living with mental health conditions in low- and middle-income countries cannot access psychological care.^5^ Providing basic care for depression in nonspecialist primary care is a global priority.^6^^,^^7^ Evidence from Africa about how best to provide this is scarce.^8^

HIV and TB have a profound impact on health in SSA. Approximately 800,000 new HIV infections were diagnosed in 2017 in Eastern/Southern Africa, bringing the total number of people living with HIV (PLHIV) in this region to an estimated 20.6 million.^9^ In 2021, TB incidence in the World Health Organization Africa Region was 2.4 million.^10^ In 2020, 73% of global HIV-associated TB infections occurred there.^11^

HIV and TB are risk factors for depression and anxiety.^12^^,^^13^ It is estimated that more than one-third of PLHIV in SSA experience depression.^13^^,^^14^ Stigma and discrimination, family life, and financial impacts intersect with sequelae of disease and treatment, posing substantial risks to mental health.^13^^,^^15^ These mental health conditions are poorly recognized and vastly undertreated.^12^^,^^13^^,^^16^^,^^17^

Treatment adherence, which is vital to good HIV and TB outcomes, involves complex and multidimensional factors and is highly linked to social pressures and mental health. Successive systematic reviews have found lower adherence to antiretroviral medication in those living with depression.^18^^–^^20^ It is clear that providing holistic care for people living with HIV and TB must include mental health support. Integrating high-quality, culturally sensitive care within routine health care systems has the potential to improve both mental health outcomes and HIV and TB treatment adherence.^21^^,^^22^ Integration of HIV, TB, and mental health into primary care is critical to improving access to this holistic care.^23^^,^^24^

Treatment adherence is vital to good HIV and TB outcomes and is highly linked to mental health. Adequate care for people living with HIV and/or TB must include mental health support.

Eswatini is a small country in southern Africa with a population of around 1 million.^25^ The Lubombo region is predominately rural and poverty is prevalent. Despite middle-income status, Eswatini is an economically unequal country, with more than 60% of the population living below the national poverty line.^26^^,^^27^ It has one of the highest HIV rates in the world at 27.4% of adults, with the highest rates in Lubombo.^28^^,^^29^ TB rates are high, with more than 4,200 new cases reported nationally in 2017, an estimated 70% in PLHIV.^30^ Public mental health care is centralized in Eswatini, with 1 psychiatrist and (at the time of this study) 2 psychologists working in 1 public National Psychiatric Referral Hospital, which is too far and too expensive to travel for many people, especially from rural Lubombo. The gap in basic mental health care nationally, while not recorded, is significant.

This study aims to assess whether the Healthy Activity Program (HAP), a psychological intervention developed in India,^31^^,^^32^ can be feasibly delivered to PLHIV and people with TB with depression within the existing primary health care system in Lubombo. It seeks to explore acceptability among health care workers and patients. This study contributes to filling the gap in knowledge of mental health intervention delivery in resource-limited settings.

## METHODS

This study used a modified version of HAP, a brief psychological treatment for depression that was developed in India by Sangath, that showed a positive effect when evaluated through a randomized controlled trial.^31^^,^^32^ The intervention involves training nonspecialist primary health care staff in the identification and causes of depression and, for those identified as having depression, using structured counseling based on behavioral activation theory with or without a referral to a doctor for further care.

Counseling consisted of 30–60 minutes sessions between individual participants and nurse counselors in the clinic with activity “homework” for participants between sessions. Key modifications from the original study were to (1) reduce the counselor’s manual size, (2) omit the patient manual (replacing vital content with handouts), (3) modify Indian-specific content, and (4) change counseling intervals from weekly to fortnightly. Modifications were made in recognition of the capacity of nurse counselors and patients, including limited literacy in the latter group, and were reviewed by individuals involved in the original study. Table 1 provides additional details about the intervention, including participants, setting, and process.^33^^,^^34^

**TABLE 1.** HAP Intervention for Depression Management Description

	**Description**
Summary and purpose	The HAP intervention for depression management in primary care includes screening, identification, counseling, and referral. The counseling was adapted from behavioral activation theory, a psychological method advocated in the World Health Organization’s Mental Health Gap Action Program. Further information is available on request. Task-shifting mental health interventions to nonspecialist health care workers is necessary to manage the gap in mental health care provision.^35^ The HAP model can be delivered in nonspecialist primary health care. In Eswatini, this was done by nurses providing HIV and TB (and other routine) care.
Procedures	A technical working group consisting of national stakeholders in HIV and TB and mental health, in addition to the participating nurse counselors, was convened to assist with the design and delivery of this study. They were held regularly throughout the study period. A modified form of the HAP, with shorter treatment duration (see methods section for modifications), was developed to be suitable and feasible for the local context, where it was conducted by clinic nurses.
Training	A 7-day training package was delivered over 2 weeks. The first week involved 4 days of training provided by a team from the Eswatini Psychiatric Team in the national Mental Health Desk Guide, a preexisting resource outlining a standard approach to the management of mental health conditions in Eswatini, provided for participating nurses not already trained on this. Thereafter, all participating nurses received 3 days of practical training in HAP delivery. A total of 16 nurses and 2 nursing assistants were trained. Feedback from training, including in this setting seldomly used role-play, was over-whelmingly positive.
Training materials	Contextualized HAP manuals National Mental Health Desk Guides Patient-held record cards Clinic record cards Activity record calendars and plans to support patients to monitor their “health activities” Paper “Patient Health Questionnaire” (PHQ)-9 forms in English and SiSwati (to screen and monitor depression) Study protocol summaries for stakeholders “8 healthy activity” posters for clinic walls All resources available here: https://comdis-hsd.leeds.ac.uk/resources/tools-tips-and-guides/. All resources were reviewed with stakeholders before the intervention was delivered. These resources were accompanied by protocols to indicate when and how participating counselors were expected to refer complex clients.
Participant eligibility criteria	Inclusion: Patients aged older than 18 years presenting for routine HIV or TB follow-up to any of the 8 participating sites. Exclusion: Pregnant patients were excluded given the different presentations, the importance of a multidisciplinary team involvement in these cases (i.e., with the maternity team) and the acute risks to mother and baby that could not be managed by the pilot team. Patients undergoing mental health treatment, as it would not have been possible to attribute changes to PHQ-9 scores due to our intervention.
Screeners	Additional nurse counselors and other clinic staff of various cadres, including expert clients and nursing assistants, received ad hoc brief training from the research assistant during clinics visits to support nurse counselors to routinely screen patients using the PHQ-9.
Counselors	Participating nurses attended 2 training sessions. All nurses were working in community or hospital TB and/or HIV clinics and had varying degrees of nursing experience. These were either all nurses at the 8 sites or those identified by training by their management because of their interest or role. A minority had any previous mental health experience or training (either in nursing college or in post-qualification in-service training). All nurses maintained their usual duties during this pilot (i.e., counseling was additional to this). Nurses that received HAP training and provided counseling are referred to as “counselors” or “nurse counselors.”
Monitoring and mentoring	Data collection, monitoring of progress or issues, and mentoring was provided by a full-time research assistant (with a community psychology background) employed for this pilot.
Supervision	Routine remote supervision of the nurse counselors was planned with a clinical psychologist initially working at the National Psychiatric Referral Hospital that could be contacted directly to discuss any complex patients. This was initially planned to be every 1–2 weeks, with the potential for acute discussion over the phone possible if needed, for example, for suicide risks.
Doctors	Doctors involved in this study received the standard non-mental health specialist medical training and were working in the regional referral hospital (with no specialist mental health care). Any further mental health education was not recorded and is highly variable.
Trainers	Training in counseling was provided by: National lead (and sole) psychiatrist (senior medical officer) Senior nurse (matron) and a psychologist working in the National Psychiatric Referral Hospital Public health doctor (in-country research lead) Research assistant.
Participant screening	Patients were screened for depression using the PHQ-9 tool. This tool was chosen as it was already being advised by the national HIV program and it had been validated in broadly comparable areas of South Africa, although not in Eswatini.^37^ It was translated into SiSwati. Where possible, patients were encouraged to self administer. Additional staff members at the participating sites were trained to approach patients to participate in the study, obtain consent, and then administer the PHQ-9 in addition to capturing basic demographic information. Consistent with the National Mental Health Desk Guide, it was agreed that patients exceeding a PHQ-9 threshold of 10 would be eligible for HAP and that those scoring 15+ or reporting suicidal thoughts would be referred for a review by a doctor to consider antidepressants, in addition to HAP.
Treatment	Patients who screened positive for depression were invited to receive HAP counseling, which is a brief psychological intervention based on “behavioral activation,” the psychological theory that espouses that mood will improve if rewarding activities are undertaken. The counseling followed 3 phases: getting to know the patient (1–2 sessions), encouraging activities (3–5 sessions), and ending well (1 session). Five to eight sessions were advised, each session 30–60 minutes. Where possible, patients were encouraged to attend the first appointment on the day of the positive screen or they were invited to reattend soon after. To build a therapeutic relationship with the counselor, each patient received a course of HAP from the same counselor. Follow-up appointments and appropriate homework tasks for the patient to complete were agreed upon at the end of each session. Initially, we planned that sessions occur at least every 2 weeks. Patients were rescreened using PHQ-9 at the beginning of each appointment. Those with high scores or suicidal thoughts (PHQ-9 score of higher than 15 or Q9 positive) would be referred to see a doctor. Once the score had fallen below 10 for 2 consecutive appointments, patients could be discharged, provided they had completed at least 5 sessions. Patients would not be provided with financial assistance to attend appointments.
Referrals	Patients were referred to the regional referral hospital (Good Shepherd Hospital) general outpatient department where they would be seen by a nonspecialist doctor. This followed routine care and procedures for referrals from clinics to doctors. These doctors would either see and discharge or, if required, refer to their mental health clinic staffed by a prescribing psychiatric nurse or to the National Psychiatric Referral Hospital.
Location	The regional health office (decentralized Ministry of Health team) selected pragmatic sample of 8 clinics in the Lubombo region of Eswatini because of nurse training or their availability to attend training, inclusion of the regional hospital and a geographical spread of health centers, and inclusion of different management and funding models (i.e., governmental, private, and faith based). The intervention was offered in 7 of the 8 sites, of which 4 were rural clinics and 3 were urban sites (1 clinic and 2 within the regional referral hospital [Good Shepherd Hospital, in its HIV and TB clinics].
Target	The target was to screen all patients attending HIV and TB clinic in all capacitated clinics (i.e., those with at least 1 HAP-trained nurse). Thereafter, all screened, consenting patients were offered 5–8 sessions of counseling every 2 weeks. Sessions lasted approximately 30 minutes in a 3-phased structure.
Tailoring	All counseling sessions were tailored to the individual, as per standard behavioral therapies. Depending on response and patient choice, the sessions could be terminated after session 5.
Modifications	Screening was not carried out systematically (see results). All eligible patients initially consented to counseling, but not all attended. In initial stages of the intervention, it was recognized that conducting sessions every 2 weeks (as originally planned) was not feasible for patients, so the schedule was changed to monthly sessions. One unwell and poorly compliant patient was provided with basic travel expenses at her counselor’s request. Supervision with the clinical psychologist was not well used, and the routine schedule dropped after only a few weeks, deferring to ad hoc supervision when needed. The reasons behind this are not fully clear and were likely a combination of nurse counselor workload, unclear chains of command, and a lack of clarity on what should be escalated (see limitations). Fidelity to these intervention protocols was monitored during regular monitoring visits by the research assistant (initially every 2 weeks, moved to every 4 weeks midway through the pilot due to challenges in travel to remote rural clinics). Fidelity to intervention content (i.e., in counseling) was assessed only in initial sessions and not with all counselors (due to logistical issues) and, while not formally graded, was deemed by the research assistant to be sufficiently adherent to our guidance. This pragmatic pilot assesses the feasibility of adherence to this guidance as part of its evaluation, and therefore this is further discussed in the results section of the main text.

Abbreviations: HAP, Healthy Activity Program.

### Evaluation

The evaluation of this study focused on investigating the feasibility and acceptability of this intervention. Feasibility was defined as the likelihood that an intervention can be recommended for further testing or scale-up and incorporates issues of demand, ease of implementation, and potential for expansion. In this context, feasibility was determined by the Ministry of Health. The study was able to make recommendations on feasibility based on the data on activity levels and interview data from nurse counselors and patients. These recommendations were presented to the Ministry of Health as a policy brief.^35^ Acceptability was defined as the “extent to which people delivering or receiving a health care intervention consider it to be appropriate, based on anticipated or experienced cognitive and emotional responses to the intervention.”^36^ This was explored from both patients’ and nurse counselors’ perspectives in the interview.

Basic quantitative service data on attendance, demographics, and Patient Health Questionnaire-9 (PHQ-9) scores were recorded and analyzed using descriptive statistics (percentage, mean, and median) in Microsoft Excel. The chi-square test was used to compare differences between categorical variables (R; version 4.2.1). Data were collected using data collection tools developed for this study to align with HIV care notes. The research assistant periodically collected the data in person from the facility-held notes.

Qualitative results were obtained via interviews with 16 patients and 6 nurse counselors purposively sampled to ensure diversity of characteristics, including gender, treatment site, and, for patients, PHQ-9 score. Patients were approached initially by their counselor, using a convenience sample, with no inclusion/exclusion criteria other than being eligible for the intervention. Nurse counselors, again using a convenience sample, were approached by the research assistant during routine study discussions, with the only inclusion/exclusion criteria that they were delivering counseling in this intervention. Interviews were undertaken at health care facilities. Patient travel costs were reimbursed (maximum of 200 Swazi emalangeni). Interviews were conducted in English by the research assistant with a siSwati translator.

The research team developed semistructured interview topic guides with a priori themes/codes and suggested probing questions. Counselor and patient topic guides were similar, both covering interviewees’ understandings of depression and experience of the study, including training (counselors only), recruitment/screening, and counseling. Patients were asked about their medical history, experiences of living with HIV and/or TB, and, if relevant, experience with antidepressants. Interview topic guides were piloted with minor language changes. Interviews occurred from May to August 2018. Interviews were audio-recorded, transcribed, and coded by 3 researchers according to the coding framework developed from interview themes. Given the similarity of counselor and patient topic guides, main codes were the same for both groups, although allowances were made for subcodes differing by group. All coded texts were checked independently by the in-country lead researcher. A framework analysis approach^37^ was used and a matrix developed with all coded texts under each theme per participant. The data under each theme were synthesized by 2 researchers, again independently checked by the lead researcher. All processes were discussed by this team to increase process validity and reliability.

### Ethical Approval

All participants gave their informed, signed consent. Identifiable information was kept in a locked cabinet. Each participant was allocated a unique number that was linked to their name on the consent form, allowing study withdrawal. Digital information was held on password-protected laptops. The study was approved by the University of Leeds ethics board (MREC16-153) and the Eswatini National Health Research Review Board (17/01/2018, no assigned number).

## RESULTS

### Quantitative

Data were collected for 9 months of follow-up: March–October 2018. In March–April, 324 patients receiving either antiretrovirals or TB medication were screened for depression using the PHQ-9 at 8 sites across Lubombo. One site withdrew early due to staffing issues. At all sites, screening of all eligible patients was encouraged, and advice was given on managing practical and logistical barriers. However, in practice, screening was done ad hoc, depending on staff capacity, availability, and engagement. All data are rounded to 2 significant figures.

Among those with a documented disease status (accounting for the different denominators), 304 of 310 (98%) patients were HIV positive and 17 of 308 (6%) had TB (of whom 9 had drug-resistant disease). Of the total 324 patients, 62 patients screened positive (PHQ-9 score of 10 or higher), indicating a depression prevalence of 19% (95% confidence interval [CI]=15.1%, 24%). Patient variables are shown in Table 2.

**TABLE 2.** Demographics of All Patients Screened and Those Who Screened Positive for Depression^a^

	**Screened, No.**	**Prevalence of Depression,**^a^ **No. (%)**	***P* Value**
Diagnosis (n=310)			<.001
HIV	303	56 (18%)	
Drug-sensitive TB	8	2 (25%)	
Drug-resistant TB	9	8 (89%)	
Gender (n=304)			.06
Male	199	46 (23%)	
Female	105	14 (13%)	
Not recorded	10		
Age, years (n=290)			.14
18–29	56	11 (20%)	
30–49	175	42 (24%)	
50 and older	59	7 (12%)	
Not recorded	34		
Education level (n=282)			.29
Primary	145	35 (24%)	
Higher	137	25 (18%)	
Not recorded	42		
Marital status (n=300)			.32
Married	136	22 (16%)	
Single	131	30 (23%)	
Divorced, separated, or widowed	33	8 (24%)	
Not recorded	24		
Employment (n=276)			<.001
Yes	115	13 (11%)	
No	162	47 (29%)	
Not recorded	48		

Abbreviation: PHQ-9, Patient Health Questionnaire-9.

^a^PHQ-9 score 10 or higher. Percentages are given based on recorded data (i.e., excluding missing records) for each variable. All data are rounded to 2 significant figures.

In this sample, the prevalence of depression was higher in men (23%) than in women (13%). The prevalence of depression was higher in those who were not currently employed (29%) compared to those employed (11%; *P*<.001). Nearly all patients screened for depression were HIV positive (93% of those screening positive, where their diagnosis was documented). The numbers for TB are too small to make any assertions, but 8 of 9 people screened with drug-resistant TB had moderate to severe depression. Of the 17 patients with TB, 12 were HIV positive, in line with national findings.^29^

**FIGURE 1:**
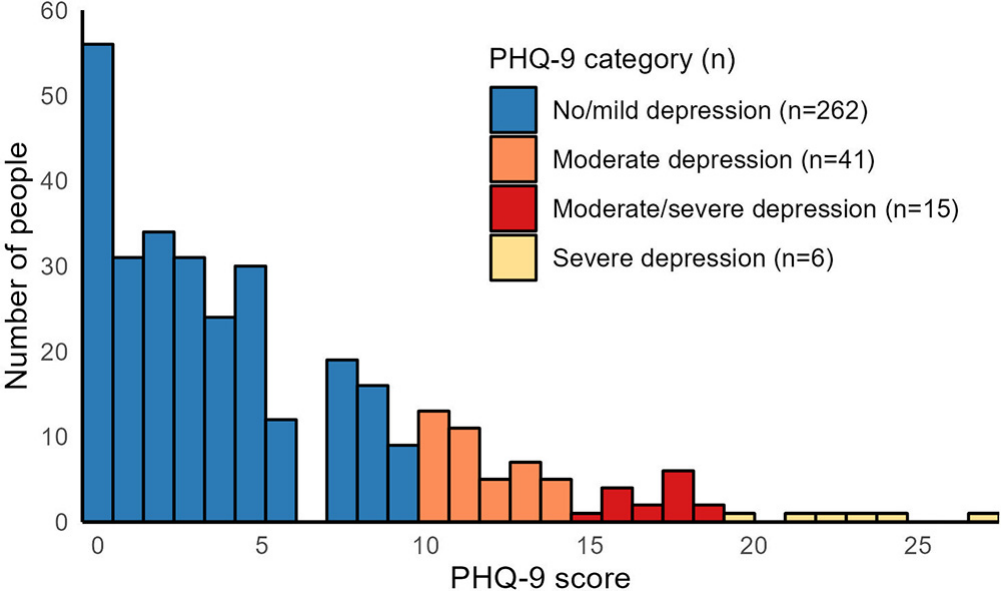
Distribution of Patient Health Questionnaire-9 Scores on Screening Among Participants in the Health Activity Program, Eswatini

All 62 patients who screened positive for depression were enrolled in HAP and accepted counseling. Two patients were thereafter withdrawn (their site withdrew). Thus, 60 patients were offered counseling. The median screening PHQ-9 score was 13 (interquartile range [IQR] 11–17). Figure 1 shows the distribution of PHQ-9 scores, with 81% having no features of depression, 13% having moderate depression, 5% having moderate to severe depression, and 2% having severe depression. Twenty-seven patients (44%) answered positively to question 9 (Q9) on suicidal ideation. Per the guideline of PHQ-9 score of 15 or higher or question 9 positive, 34 patients (55%) should have been referred, but only 13 were recorded as having been referred (11 to a doctor, with 5 prescribed antidepressants).

**FIGURE 2:**
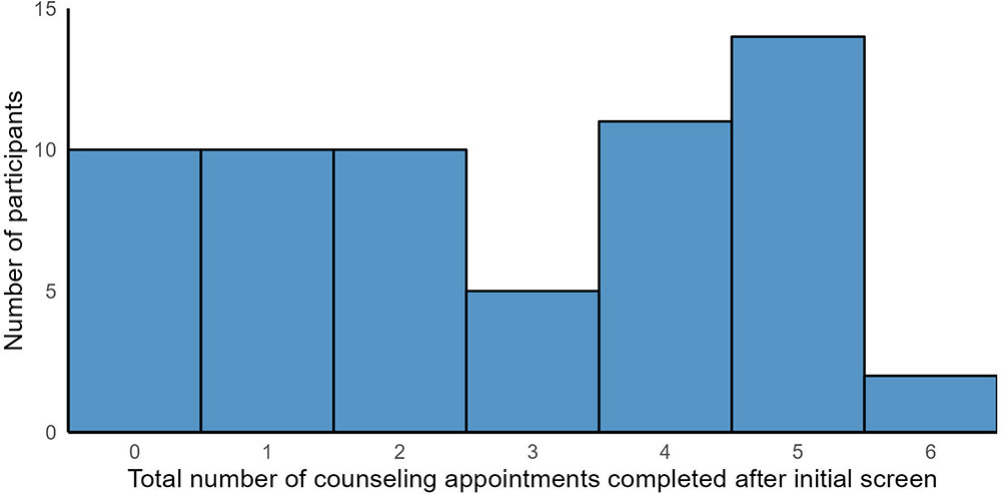
Distribution of the Total Number of Healthy Activity Program Counseling Sessions Attended, per Participant, Eswatini

Of the 60 patients, 52 attended at least 1 counseling session. Figure 2 shows the variation in the total number of HAP sessions attended by each client, with the median number of sessions being 3 (IQR 1–5). The median interval between sessions was 29 days (IQR 24.5–45) (excluding between screening-counseling session 1 (S1) as this was 0 in 87% of patients). Mean session length was 27 minutes. Early in the evaluation process, the session interval frequency was modified to be monthly (from fortnightly) to coincide with HIV and TB medication collection and because shorter intervals proved to be unrealistic due to patient transport, time, and finances.

### Qualitative

Table 3 shows nurse counselor characteristics, and Table 4 shows patient characteristics.

**TABLE 3.** Characteristics of Nurse Counselors Interviewed

	**No. (N=6)**
Gender	
Male	2
Female	4
Setting of interview and counseling provision	
Community clinic	4
Hospital clinic	2

**TABLE 4.** Characteristics of Patients Interviewed

	**No. (N=15)**
Gender	
Male	5
Female	10
Setting of interview and counseling provision	
Community clinic	9
Hospital clinic	6
Baseline PHQ-9 score	
10–14	9
15+	6
Employment status	
Employed	1
Unemployed	14
Diagnosis	
HIV	11
Only TB	0
HIV and TB	4
Marital status	
Married	6
Unmarried	9

Abbreviation: PHQ-9, Patient Health Questionnaire-9.

Nine codes were identified a priori, each comprising 2–7 subcodes. An “other” code allowed for emergent themes. All codes are summarized:

1. Intervention context
  - Understanding depression
  - HIV and/or TB and depression
  - Existing support and treatment
  - Barriers/facilitators to scale-up
2. Intervention experiences
  - Screening and diagnosis
  - Referrals and antidepressant use
  - Counseling

Interview findings are discussed below using this framework. Counselors and patients expressed similar views, albeit often using different terms, reflecting their joint sociocultural experiences but different levels of medical education and different experiences of the intervention. Where views diverged, this is made clear in the text below.

#### Intervention Context

Depression, while often not named (with no direct translation into SiSwati), was recognized by counselors and patients—both in patients/themselves and in their communities. Multiple factors in this setting, including especially HIV, were recognized to have significant impacts on well-being. Despite this, both groups identified few or no prior strategies or management for depression in their settings, reinforcing concerns about a treatment gap. Counselors identified contextual barriers to scaling up this intervention, including workload, time, travel, and availability of complementary services.

Both counselors and patients identified few or no prior strategies or management for depression in their settings, reinforcing concerns about a treatment gap.

All interviewees understood depression as affecting mood and function. Counselors emphasized biological/circumstantial causes. Patients discussed their feelings, such as hopelessness, “fail to think straight,” and “heavy load on my chest.”

Suicide was mentioned by all but 1 counselor. Patients did not use this term, yet discussed death associated with depression.

*Before [counseling] my life span was shortened.* —Patient

*If it wasn’t for the study maybe I would have been dead by now”* —Patient

All interviewees discussed the psychological impact of having HIV and/or TB.

*It really destroys the individual, mentally, emotionally, social [sic] and otherwise.* —Nurse counselor

Patients discussed ostracism.

*No one would touch anything from me, no one would eat with me, it was a very bad life.* —Patient

Social isolation, stigma, and family issues were common, including suggestions of abuse (the latter discussed only by female participants)

*He no longer treats me like he used to in the past, probably it’s because he knows I am positive…he is abusive.* —Patient

A recurrent fear was that people would know or find out through medication, side effects, or visible symptoms.

*Everyone now knows that [the patient] ha[s] HIV and this makes them depressed.* —Nurse counselor

This was discussed concerning nonadherence alongside depression itself.

*Sometimes you feel like even not even taking your medication.* —Patient

Regarding existing support and treatment, all counselors expressed pre-pilot challenges in managing depression.

*It was so frustrating because you would see that this person has got a problem but you did not have skills…to help them.* —Nurse counselor

They reported lack of time, training, protocols, and systems.

Patients expressed concerns about confidentiality and cultural and social norms around discussing mood.

*In my culture it is not allowed to disclose the problems you have in your household with your husband.* —Patient

*I never like to tell people my problems because others tend to judge you…if you tell them your problems they start telling others.* —Patient

Overall, both groups felt this service should be scaled up, but logistics prevented this. Barriers to scale-up included staff capacity, privacy, space, patients not attending sessions, and patient finances affecting health care access (travel/time off work). Time was a recurrent issue for counselors and patients.

*It takes a lot of time for them to go through that process … patients will be…impatient because they will be wanting to go because they've taken so much time in the clinic going through the process of refilling their medication and now you want to go for a forty-minute session of counseling…* —Nurse counselor

*I’m a seasonal worker so I cannot come every month it might disturb my job.* —Patient

Both counselors and patients felt the program should be scaled up, but logistical barriers prevented this.

To facilitate scale-up, counselors recommended further task-shifting/increasing workforce capacity, as well as sensitizing and engaging stakeholders and the public.

Counselors also identified that some stressors identified by participants (e.g., intractable financial problems) were less suited to a problem-solving approach.

*[Patients] needed financial assistance…we try…but we could not help them* —Nurse counselor

Other problems (e.g., previous traumatic experiences) were felt to be too complex to be addressed using the HAP approach alone and were likely to require additional specialist psychological input.

*Our clients here [have] problems which are a bit…difficult and HAP doesn’t really get into them so deep.* —Nurse counselor

#### Intervention Experiences

Counselors and patients indicate that they found screening, diagnosis, and counseling broadly acceptable. However, they expressed some concerns about the need to repeat the PHQ-9 assessment at each session. Counselors appreciated the clear guidance on when to refer patients (although referral pathways remained an issue as outlined in the quantitative data and discussed later).

Generally, both counselors and patients found the PHQ-9 easy to use and helpful in identifying depression. Patients reported diagnosis as “shock/unexpected” sometimes followed by anticipation.

*It made me have something to live for, it made it clear that there was help on the way*. —Patient

*They gave me hope.* —Patient

Some patients struggled with the screening.

*I was hiding the feeling…I didn’t like them asking me what is wrong, I wanted it to be a secret*. —Patient

*You feel like you are being exposed*. —Patient

Counselors discussed logistical issues, including lacking capacity to systematically screen patients due to workload, lack of space and privacy, and issues with language.

*Most clients felt it’s a bit difficult for them even if they were siSwati speakers.* —Nurse counselor

Regarding referrals, counselors responded favorably to the referral guidelines, yet they reported challenges in monitoring whether patients attended, with no mechanism to confirm referral.

In both patients and counselors, there was increased awareness of depression. Counselors found counseling fulfilling. All patients and 3 counselors reported mood improvements in patients. Both patients and counselors deemed the use of “behavioral activation” helpful, with patients suggesting activities, but some required more sociocultural sensitivity.

*What surprised me is when the counselor told me to go and exercise because how was I going to exercise as I normally work*. —Patient

*[Patients] would come back without doing the given tasks stating that they thought they were not important.* —Nurse counselor

All patients interviewed reported mood improvements after attending counseling.

Counselors suggested some positive impacts on medication adherence.

Regarding using the PHQ-9 at each session to monitor response, counselors expressed concerns.

*Patients become used to the tool and they lie.* —Nurse counselor

In general, patients found the tool useful, but negative responses existed, including concerns about exhaustion and repetition.

## DISCUSSION

This study contributes important evidence to the World Health Organization’s call to improve mental health for all^38^ in a particularly at-risk group in an understudied setting. It furthers unde-standing of the integration of mental health into primary care, in addition to advocating for a low-resource, replicable method to do this.

This study found depression to be common among PLHIV and people with TB in this context. Our analysis indicates that, while there are some potential benefits to HAP in this setting, there are caveats in terms of feasibility and acceptability for patients and the health system.

### Feasibility and Acceptability

Qualitative results suggest acceptability of this program. However, it should be noted the lack of qualitative evidence from patients not attending counseling weakens this assertion. There were positive accounts of HAP on patients’ lives, mood, and well-being. However, the quantitative results indicate that patients did not attend a suggested course (5–8 sessions) in 9 months of follow-up. While a degree of attrition may be expected in psychological therapies, this is potentially excessive. This observation puts into question both the feasibility and acceptability of the 9-month course duration in this setting. In future practice or research, shorter courses could be trialed, and patients could be explicitly asked beforehand of the potential barriers affecting attendance so that potential mitigating factors could be put in place. While this was unfortunately not possible in this pilot, future research should attempt to follow up with patients no longer attending counseling.

There were concerns about the validity and reliability of the PHQ-9 used as a screening tool in this way, where it is not validated. Median PHQ-9 scores dropped sharply after 1 session, suggestive of a factor other than treatment effectiveness. While this study does not aim and is not powered to assess effectiveness, these findings raise questions about the use of this tool, despite its validity in similar settings.^39^^–^^41^ Interviews with counselors introduced speculation as to whether initial scores were artificially inflated due to misunderstandings (there is no term for depression in siSwati, and questions proved complex to translate) or transient interference of HIV and/or TB symptoms or medication side effects, thus causing the PHQ-9 to improve rapidly at later sessions. This is conjecture; however, these concerns align with previous evidence, where a risk of false positives in screening tools for depression among PLHIV was recognized in a review across Africa.^42^ This complicates any assessment of feasibility, as it is unclear whether our patient selection was correct. Alternatively, there were concerns that patients purposefully lowered their scores to please the counselor or expedite the end of the sessions, with implications on acceptability. Such concerns could be addressed with a randomized controlled trial, which we recommend for this context.

Concerns expressed by health care professionals about the sustainability of this intervention centered around their capacity, space, and workload, reinforced by 1 site withdrawing early in the study. Nurses have little spare capacity and many competing priorities, inhibiting their ability to deliver 30-minute sessions while providing care to other patients. A feasibility study conducted in South Africa with a similar intervention for people living with HIV and/or diabetes found similar rates of feasibility and acceptability for “designated” (alongside usual duties) and “dedicated” (sole responsibility) health care workers.^43^ A further small study (n=14) in South Africa suggested acceptability and a positive impact on adherence to this intervention but again concerns around feasibility (in this case, around provider fidelity to the intervention).^44^ A study in Zimbabwe implemented more supervision, mentoring, and allocated time for their nurse counselors for this intervention and indicated better treatment adherence.^45^ These additional support measures may improve feasibility, acceptability, and patient adherence. None of these relatively small studies are conclusive. However, they indicate that integration is feasible into routine care and that a careful balance of workforce support and health care support is required. These studies also involved other/lay health cadres, which was a suggestion raised by our counselors and one we advocate to pursue in further research.

Issues around referrals could be interpreted in 2 ways: (1) patients did not want or could not attend; or (2) referral pathways and quality accessible secondary mental health care were lacking. These indicate a lack of acceptability (by patients) and/or feasibility (insufficient mental health infrastructure).

### Prevalence

Almost one-fifth of those with HIV and/or TB who were screened were diagnosed with depression, with a high proportion of suicidal ideation. These small numbers should be interpreted cautiously yet earnestly, given alignment with existing evidence^46^ and reinforcement by qualitative results. Having HIV and/or TB and poverty, traumatic events, familial and community pressures intersect, placing this population at a high risk of mental disorders. Our findings support the view that stigma related to HIV and/or TB increases the risk of mental disorders and adversely affects HIV^47^ and TB^48^ outcomes, findings replicated in Eswatini.^49^

### Recommendations for Policy and Practice

This pilot study shows substantial unmet needs and potential benefits of this approach, as well as feasibility concerns. Training nonspecialist health care staff in common mental disorders is recognized globally as essential to managing the treatment gap,^38^ and integration with HIV and TB care is critical.^12^^,^^50^ But there are meaningful human resource constraints. Implementation needs to be realistic, sensitive, and pragmatic. In an integrated service and in the absence of additional staff, there is an opportunity cost to other services that may be inappropriate. Wider education and community engagement are necessary to reduce stigma and raise the profile of mental disorders in a manner that supports acceptance of talking therapies. These findings broadly align with similar research.^51^

### Limitations

This was a small pilot in line with the values and aims of implementation research; it was pragmatic, operational, and collaborative.^52^ There are benefits to this methodology, but in addition to its relatively small scale, limitations warrant discussion. In all stages of this study, this approach impeded ideal implementation, including participating clinic and nurse selection, lack of systematic screening, and an inability to follow up on missing information that affected data completeness. The low number of patients referred to a doctor was concerning and a major limitation of this study, especially given their higher risk of harm. This was flagged during the pilot, yet there are no longitudinal data to assess their follow-up or management. We were not able to follow up these patients, given a lack of contact details and reporting delays. Upon questioning, many counselors reported that patients refused referral due to cost of travel or unspecified reasons.

Interviews were only feasible with patients who attended counseling; hence, the experiences of those not attending were not captured and limited our conclusion on acceptability. Informal discussions with health care staff suggest nonattendance was due to a combination of factors, including patients’ lacking time, lack of cultural norms around counseling, and organizational issues meaning counselors were not aware patients were present, although this is conjecture. For future longer-term evaluation of this intervention, this needs to be a key focus of investigation and follow-up.

Fidelity to intervention delivery over time was insufficiently assessed in this pragmatic pilot, and this weakens our understanding of both acceptability and feasibility. The mean session length (27 minutes) indicates some alignment with the suggested timings, and interviews indicate some fidelity. However, it is difficult to adequately assess how HAP counseling differed from the more unstructured support routinely provided by nurses in these settings, especially over time. This warrants further monitoring in future studies.

Finally, the clinical psychologist was not effectively or appropriately used, with reasons not fully understood. Supervision and mentoring are important elements of task-shifting and may have improved safety, quality, fidelity, and feasibility.

## CONCLUSION

This intervention is promising and generates useful recommendations on feasibility. Real-life application is tempered by health system resources and capacity, workforce availability, and training constraints. Patients’ ability to complete a course is limited by travel costs, time, and social barriers—all issues not unique to Eswatini. This small study is an incremental step to understanding the challenges, complexities, and successes of implementing a contextualized mental health package in a setting with little alternative access to care for individuals with common mental health problems but who have great need.

## Notes

**Peer Reviewed**

**First published online:** March 29, 2023.
